# Evaluating two respiratory correction methods for abdominal PET/MRI imaging

**DOI:** 10.1186/s40658-022-00430-w

**Published:** 2022-01-31

**Authors:** Weiwei Ruan, Fang Liu, Xun Sun, Fan Hu, Tingfan Wu, Yongxue Zhang, Xiaoli Lan

**Affiliations:** 1 Department of Nuclear Medicine, Union Hospital, Tongji Medical College, Huazhong University of Science and Technology, No. 1277 Jiefang Ave, Wuhan, 430022 China; 2 Hubei Province Key Laboratory of Molecular Imaging, Wuhan, 430022 China; 3 GE Healthcare, Shanghai, 201203 China

**Keywords:** PET, MRI, Respiratory motion correction, Standard uptake value, Abdomen

## Abstract

**Background:**

To evaluate two respiratory correction methods for abdominal PET/MRI images and further to analyse the effects on standard uptake values (SUVs) of respiratory motion correction, 17 patients with 25 abdominal lesions on ^18^F-FDG PET/CT were scanned with PET/MRI. PET images were reconstructed using end-expiratory respiratory gating and multi-bin respiratory gating. Meanwhile, full data and the first 3 min and 20 s of data acquired both without respiratory gating were reconstructed for evaluation. Five parameters, including the SUV_max_ and SUV_mean_ in the lesions, the SUV_mean_ and standard deviation (SD) in the background, and the signal-to-noise ratio (SNR), were calculated and used for statistical comparisons. The differences in multi-bin respiratory gating and reconstruction of full data, relative to the reconstruction of the first 3 min and 20 s of data acquired, were calculated.

**Results:**

Compared with PET/CT, the longer scanning time of abdominal PET/MRI makes respiratory motion correction necessary. The multi-bin respiratory gating correction could reduce the PET image blur and increase the SUV_max_ (11.98%) and SUV_mean_ (13.12%) of the lesions significantly (*p* = 0.00), which was much more effective than end-expiratory respiratory gating for abdominal PET/MRI. The added value of SUV_max_ caused by respiratory motion correction has no significant difference compared with that caused by count loss with the correction (*p* = 0.39), which was rarely reported by previous studies.

**Conclusion:**

Based on the current parameters, the method of multi-bin respiratory gating was more effective for respiratory motion correction in abdominal PET/MRI in comparisons with the method of end-respiratory gating. However, the increased noise in gated images, due to the fact that PET data get discarded, is partly responsible for the increase in SUV_max_.

## Background

Positron emission tomography (PET) uses radiotracers to image functional changes for detecting diseases. It is usually more sensitive and can detect disease foci earlier than structural imaging [1]. PET is critical for the staging and assessment of treatment response in many cancers. The standardised uptake value (SUV) is usually used in PET [2, 3]. Accuracy of uptake measurement is vital.

Respiratory motion during scanning can blur images, affect the SUVs of images, and cause bias in its measurement [4]. Many methods for correcting respiratory motion in PET imaging have been reported [5–7]. The key thinking has been to isolate the quiescent period based on the patient’s measured respiratory waveform (amplitude or phase). For example, end-expiration respiratory gating is based on the observation that the patients usually have a quiescent period after end-expiration during the breathing cycle. Only the counts acquired during the quiescent period were considered valid and used for reconstruction [8, 9]. However, these respiratory motion correction methods are seldom used in routine clinical scanning. For PET/CT, whole body scanning usually requires only 2 min per bed position, and the speed of scanning has been increasing with the improvement of PET detectors [10], which has limited the effects of respiratory motion.

PET/MRI has been in clinical use for over 10 years [11]. PET/MRI has many advantages over PET/CT, especially improved soft tissue contrast and the elimination of ionising radiation from CT. The combination of PET and multi-sequence MRI, that is, T1-weighted (T1WI), T2-weighted (T2WI), and diffusion-weighted imaging (DWI), provides more information for diagnosis. However, the characters of multi-sequences scanning for PET/MRI decided the scanning time much longer than that for PET/CT. Even for single-site scanning, the time often approximates 10 min. With this increased scanning time, the effects of respiratory motion on PET images will increase, making respiratory motion correction necessary.

**Fig. 1:**
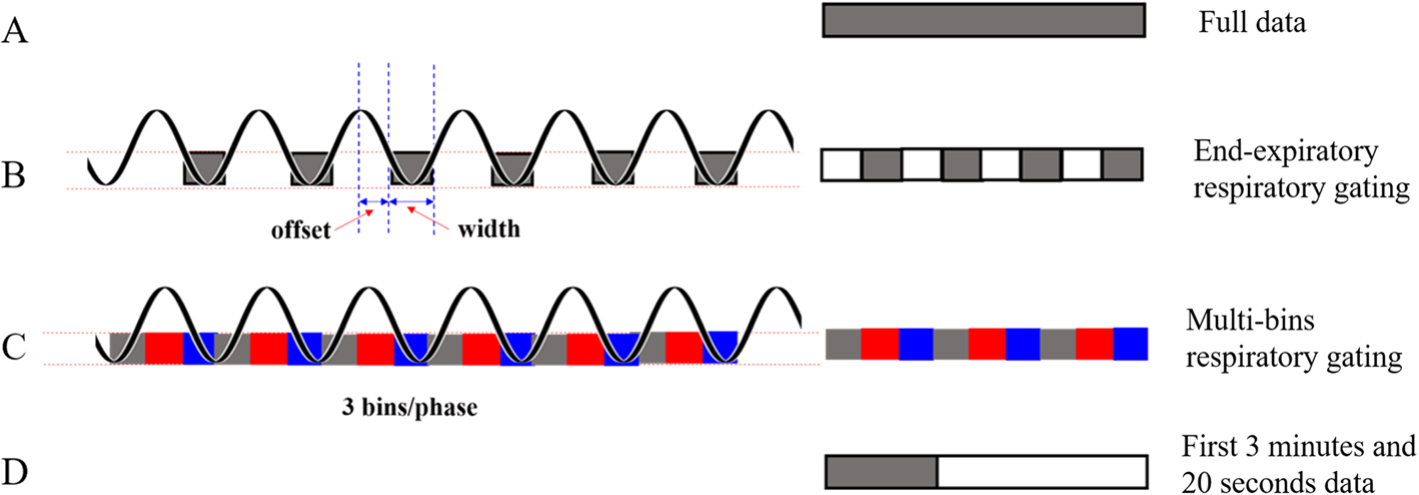
Four abdominal PET/MRI reconstruction methods. The left side of the figure shows the ideal respiratory cycle. The right shows diagrams of the four reconstruction methods

The most commonly utilised respiratory motion correction technique is end-expiration respiratory gating. The system in our PET/MRI (Q.Static, GE Healthcare) uses this quiescent part of the respiratory cycle. End-expiratory respiratory gating has two key parameters, offset and width [8], and it relies on the frequency and amplitude of the respiratory cycle (Fig. 1). The offset determines the initial point for the quiescent phase, and the width represents the proportion of the quiescent time in the respiratory cycle. Grootjans et al. used a pressure sensor integrated in an elastic belt placed around the patient’s thorax and reconstructed the images with 50%, 35%, and 20% of acquired PET data (respiratory cycle) for respiratory motion correction. Their optimal respiratory-gated images were reconstructed with a duty cycle of 35% [12]. Hope et al. evaluated the effects of end-expiratory respiratory gating for liver PET images using six patients’ ^68^ Ga-DOTATATE PET/MRI scans. Their results suggest that SUV_max_ improved significantly compared with ungated data, which used the 50% of the respiratory period from accepted breath-holds for the final reconstruction [13]. Another respiratory motion correction technique is multi-bin respiratory gating **(**Fig. 1), which is used to replay gated images to eliminate respiratory motion by subdividing the data into bins (phases) to better visualise the respiratory cycle. And the amplitude of respiratory cycle would not be the basis for discarding the PET counts or not. Previous study mainly focused on developing new methods and compared them with the results of static PET on PET/CT [8, 13, 14]. For the PET/MRI, it was unknown for the comparison of effects on PET quantitation between the end-expiratory respiratory gating and multi-bin respiratory gating for respiratory motion correction.

The SUV_max_ calculations are sensitive to noise. Fewer scan time will increase the image noise, causing the SUV_max_ increasing falsely, which has been demonstrated in previous works [15]. For the PET reconstruction with respiratory motion correction, there are two phenomena affecting the SUVs. On the one hand, the reduction of respiratory blurring will increase measured SUVs [16]. On the other hand, the respiratory motion correction isolated the PET counts with consistent respiratory amplitude or phase and dropped others, which results in the fewer PET counts and increased the image noise. However, comparisons of these two aspects and their effect on SUVs have rarely been reported.

Here, we compared the two respiratory motion correction methods, end-expiratory respiratory gating and multi-bin respiratory gating in abdominal PET/MRI. The full data without any respiratory gating were reconstructed as a reference. And further, to avoid the influence of time, the data of the first 3 min 20 s without respiratory gating were also reconstructed. The data were 1/3 of the full data, which was consistent with the amount of data used for final reconstruction with respiratory motion correction. The SUV both in lesions and the background was measured and calculated for comparisons and evaluation. The aim of this study was to evaluate two respiratory motion correction methods and further explore how to balance respiratory motion and the lost counts due to respiratory motion correction in abdominal PET/MRI.

## Methods

### Patients

This was a retrospective study. ^18^F-FDG PET/MRI was performed in patients with lesions with obvious high uptake in the abdomen on ^18^F-FDG PET/CT images. After the PET/CT scan, the patients underwent a PET/MRI scan without extra injection of ^18^F-FDG. The exclusion criteria were any contraindication against MRI scanning [17]. Patients demonstrating obvious body movement during the PET/MRI scanning were also excluded. Finally, 17 patients with 25 obvious abdominal lesions were included, which mainly involved the liver, pancreas and peritoneum lesions. The study was approved by the Ethics Committee of Tongji Medical College, Huazhong University of Science and Technology. The need for written informed consent was waived.

### Imaging protocol

Patients underwent ^18^F-FDG PET/MRI imaging of the abdomen in a PET/MRI scanner (3.0 T, SIGNA TOF-PET/MRI, GE Healthcare). Before scanning, respiratory bellows were fastened to the abdomen for respiratory monitoring.

PET acquisition was conducted in respiratory gating mode, and all PET data were acquired and stored by list-mode data. The scanning time was 10 min. During the PET acquisition, four common abdominal MRI sequences were acquired: (1) MRI-based attention correction (MRAC) sequence based on Dixon water–fat separation; (2) axial T_2_WI with fat suppression, which were free-breathing because of the breathing trigger; (3) axial DWI with fat suppression, which were free-breathing due to the breathing trigger; and (4) three-dimensional liver acceleration volume acquisition (LAVA) T_1_WI, during which the patient was asked to hold their breath for 18 s during scanning.

### PET reconstruction

PET images were reconstructed in four ways (Fig. 1). The first method used full data for reconstruction without respiratory gating **(**Fig. 1A). The end-expiratory respiratory gating mode used only the data acquired at end-expiration, in which the offset and width were both set at 33% based on the breathing amplitude, which were close to the 35% suggested by previous reports [12] **(**Fig. 1B). The multi-bin respiratory gating mode processed data that were divided into three phases for every respiratory cycle. As shown in Fig. 1C, the same colour represented the same respiratory phase. Therefore, for the PET reconstruction with multi-bins respiratory gating correction, three groups of abdominal PET images could be obtained, which were corresponding to the three phases. We observed that there almost have no different for the three groups of PET images quantitation. We always used the PET images corresponding to the first respiratory cycle for the following comparisons and analysis. Therefore, the amount of data used for PET reconstruction was only 1/3 from the full data, which was consistent with the end-expiratory respiratory gating mode, and it was equivalent to the data from only 3 min 20 s scanning (Fig. 1D). To avoid the influence of time, for the fourth way, the data of the first 3 min 20 s without respiratory gating were also reconstructed. Except for the difference in respiratory motion correction, the other construction parameters were all the same: the ordered subsets expectation maximum (OSEM) algorithm with TOF technique was used for construction, FOV = 60 cm × 60 cm, Matrix = 192 × 192, Filter Cut-off = 3.0 mm, Subsets = 28, Iterations = 2. The attenuation correction of PET was based on MRI data and combined tissue segmentation and template matching methods [18].

### Qualitative evaluation of SUV

Quantitative calculations were used to evaluate PET image quality, including the following five parameters: the SUV_max_ and SUV_mean_ of lesions, the SUV_mean_ and standard deviation (SD) of the background, and the signal-to-noise ratio (SNR) for each lesion. The SNR was calculated as follows [6]:

1 $${\text{SNR }} = {\text{SUVL}}/{\text{SD}}$$

where “SUVL” represents the SUV_mean_ of lesions and SD represents the standard deviation of the background. SNR is a common parameter used to evaluate the quality of PET images. The SUV_max_ of the background was not used here, due to the uncertainty in single-pixel background measurements caused by electronic and environmental noise [19].

Further, we calculated the differences among all parameters (except SUV_mean_ of the background), that is, reconstruction of full data without respiratory gating and multi-bin respiratory gating groups relative to reconstruction of the first 3 min and 20 s of data acquired without respiratory gating.

The 3D volume of lesions was calculated using 42% threshold segmentation. The region of interest for the background was defined as a 3-mm-diameter sphere. All work was completed in GE AW workstation 4.6 (GE Healthcare).

### Statistical analysis

First, we aimed to compare the differences among the five quantitative parameters in the four groups to evaluate the statistical significance. The homogeneity of the variance distribution of the data for every parameter was tested with the Levene statistic. After verifying the variance distribution to be homogeneous, the following statistical analysis was completed by two-way analysis of variance of randomised block design.

Second, we aimed to analyse whether the results of differences in reconstruction of all data without respiratory gating and multi-bin respiratory gating groups relative to the reconstruction of the first 3 min and 20 s of data acquired without respiratory gating group were significantly different. The variance distribution was also tested with the Levene statistic. If the variance was homogeneous, the independent *t*-test was used for the following analysis. Otherwise, the Mann–Whitney *U*-test was used.

For the homogeneity of variance test, a *p* > 0.05 was considered homogeneous. For the mean value testing, *p* < 0.05 was considered a statistically significant difference. Statistics were analysed using SPSS Statistics (version 24; IBM).

## Results

Table 1 lists the mean values and SDs for the overall data. The statistical testing for variance homogeneity suggested that variances for every parameter in the four groups were all homogeneous (*p* > 0.05). Therefore, it was reasonable to use two-way analysis of variance of randomised blocks for the statistical comparisons to test the paired data in the four groups and obtain statistical results. The different percentages between pairs of groups were also calculated and are shown in Table 1.

**Table 1:** Mean and standard deviation (SD) for the five parameters in all four groups are listed

	SUV_max_ (Lesions)	SUV_mean_ (Lesions)	SUV_mean_ (Background)	SD (Background)	SNR
*Mean ± SD*					
^a^Method 1	8.34 ± 3.02	4.81 ± 1.96	1.69 ± 0.35	0.28 ± 0.18	22.81 ± 15.28
^b^Method 2	8.62 ± 3.23	4.98 ± 2.07	1.68 ± 0.34	0.37 ± 0.21	16.94 ± 11.07
^c^Method 3	9.83 ± 3.67	5.73 ± 2.36	1.69 ± 0.33	0.37 ± 0.22	19.36 ± 12.03
^d^Method 4	8.84 ± 3.30	5.11 ± 2.15	1.70 ± 0.34	0.35 ± 0.20	18.47 ± 13.43
*Variance testing*					
*F*-value (*p*-value)	0.96 (0.42)	0.87 (0.46)	0.02 (0.99)	1.06 (0.37)	0.91 (0.44)
*Difference percent (p-value)*					
2 vs 1	2.90% (0.22)	3.09% (0.25)	− 0.53% (0.41)	36.68% **(0.00)**	− 23.52% **(0.00)**
3 vs 1	18.15% **(0.00)**	19.70% **(0.00)**	0.46%(0.76)	36.98% **(0.00)**	− 11.30% **(0.00)**
4 vs 1	5.76% **(0.03)**	5.99% **(0.04)**	0.63%(0.55)	31.41% **(0.00)**	− 18.00% **(0.00)**
2 vs 4	− 2.28% (0.37)	− 2.35% (0.35)	− 1.01%(0.16)	4.53% **(0.03)**	− 5.93% (0.08)
3 vs 4	11.98% **(0.00)**	13.12% **(0.00)**	− 0.10%(0.78)	5.05% **(0.03)**	9.39% (0.30)
3 vs 2	15.41% **(0.00)**	17.54% **(0.00)**	1.04% (0.26)	0.64%(0.96)	19.18% **(0.01)**

For obvious comparisons, the percent difference between pairs of groups was also calculated and shown

*p* < 0.05 was considered a statistically significant difference between groups, which were shown in bold

^a^The reconstruction of full data without respiratory gating

^b^The reconstruction of end-expiratory respiratory gating

^c^The reconstruction of multi-bins respiratory gating

^d^The reconstruction of first 3 min and 2 s data without respiratory gating

For the SUV_max_ and SUV_mean_ of lesions, the values in the multi-bin respiratory gating group were largest. The SUV_max_ and SUV_mean_ increased 11.98% and 13.12%, respectively, compared with the reconstruction of the first 3 min and 20 s of data acquired without respiratory gating group (*p* = 0.00). These values in the end-expiratory respiratory gating group showed no significant difference with those in the reconstruction of the first 3 min and 20 s of data acquired the group without respiratory gating (*p* = 0.37). The values for the SUV_max_ and SUV_mean_ of lesions were lowest in the group with reconstruction of full data without respiratory gating.

**Fig. 2:**
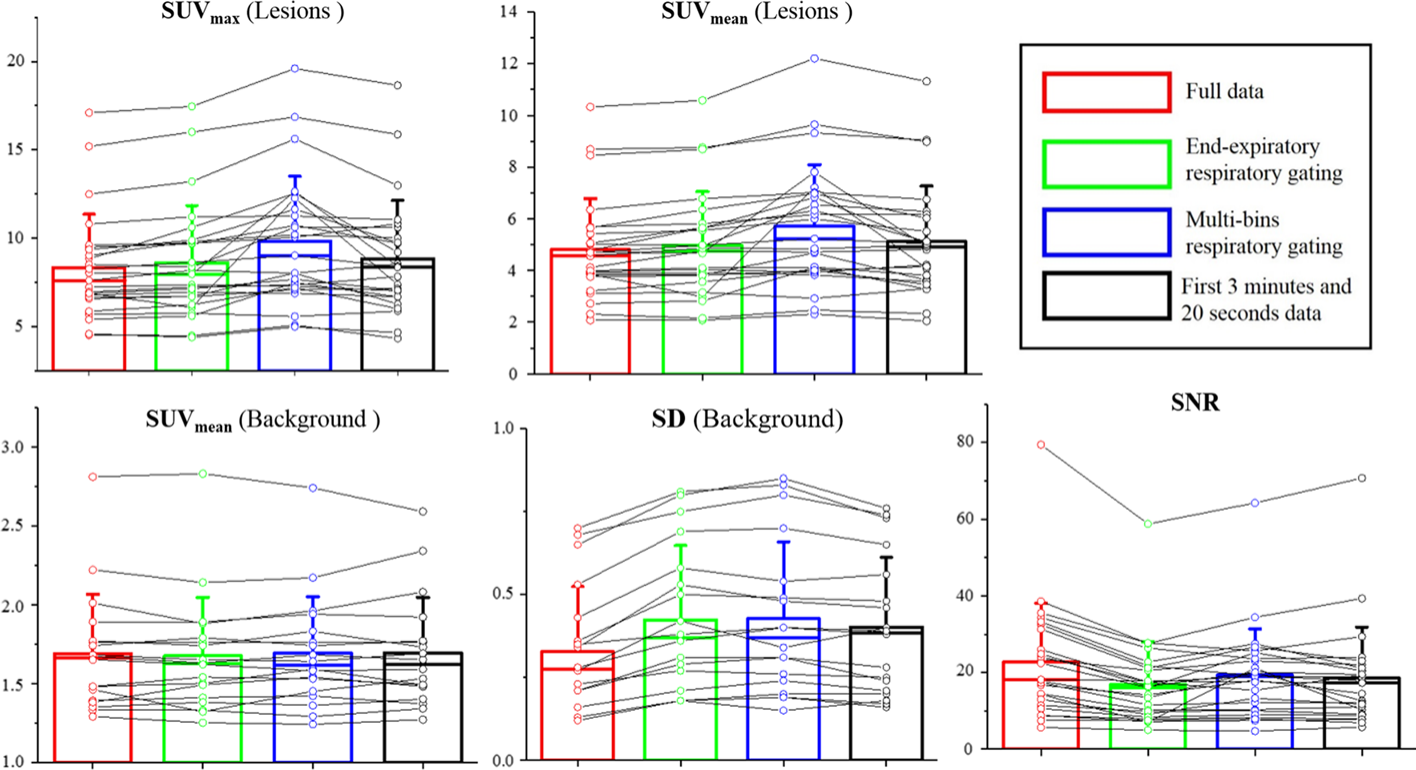
Means and standard deviations of the five parameters in the four groups. Different colours represent different groups, as shown in the top right corner. The detailed data are also shown by dots and the connected lines for visual paired comparisons

For the SUV_mean_ of the background, there were no significant differences among all four groups. For the SD of the background, the values in the reconstruction of full data without respiratory gating were the lowest, which means the noise level was lowest if the PET was reconstructed in this way. The SDs in the multi-bin respiratory gating and end-expiratory respiratory gating groups were both slightly larger than those in the reconstruction of the first 3 min and 20 s of data acquired without respiratory gating group (4.53% and 5.05%, respectively), which means the methods with respiratory gating both increased background noise. Although the SNR in the group with reconstruction of full data without respiratory gating was the highest, the value in the multi-bin respiratory gating group also improved by 19.18%, which was significant (*p* = 0.01) compared with that in the end-expiratory respiratory gating group. The paired comparisons of the datasets are shown in Fig. 2. The results are consistent with those shown in Table 1.

**Fig. 3:**
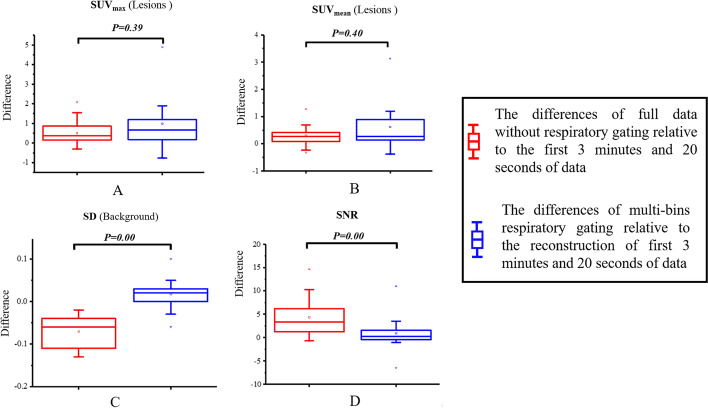
Comparison of difference among the reconstruction of full data without respiratory gating relative to the reconstruction of the first 3 min and 20 s of data (red boxes), the reconstruction of multi-bins respiratory gating relative to the reconstruction of first 3 min and 20 s of data (blue boxes), for four parameters, the SUV_max_ (**A**) and SUV_mean_ (**B**) of lesions, the standard deviation of background (**C**) and SNR (**D**). The corresponding statistical results were also shown

Differences shown in Table 2 were calculated by subtracting the values between the reconstruction of full data without respiratory gating and reconstruction of the first 3 min and 20 s of data acquired without respiratory gating, and between multi-bin respiratory gating and reconstruction of the first 3 min and 20 s of data acquired without respiratory gating. Initial variance testing was also conducted for every parameter. The different SUV_max_ and SUV_mean_ values in the lesions showed that variance was not homogeneous (*p* = 0.006 and 0.005, respectively). In subsequent analysis, the Mann–Whitney U test was used, and the differences in SUV_max_ and SUV_mean_ were not significant (*p* = 0.39). The variances of the other two parameters were homogeneous (*p* > 0.05). The independent *t*-test was used, and the results suggested that the SD and SNR were all significantly different between the two difference calculation groups (*p* = 0.00). The corresponding results are shown in box plots in Fig. 3, which displays the comparisons of the two groups.

**Table 2:** Lists of the differences for the reconstruction of full data without respiratory gating and multi-bin respiratory gating relative to the reconstruction of the first 3 min and 20 s of data acquired without respiratory gating, including the mean value, SD and the corresponding statistical results

	Lesions		Background	
	SUV_max_	SUV_mean_	SD	SNR
*Difference (Mean ± SD)*				
*A	0.5 ± 0.58	0.30 ± 0.35	− 0.07 ± 0.04	4.34 ± 3.99
*B	0.99 ± 1.44	0.61 ± 0.88	0.02 ± 0.04	0.89 ± 3.82
*Variance testing*				
*F*-value (*p*-value)	8.25 (0.006)	8.54 (0.005)	0.52 (0.475)	0.66 (0.42)
*Mean value testing*				
*p*-value	*Z* = − 0.85 *p* = 0.39	*Z* = − 0.84 *p* = 0.40	0.00	0.00

*A and *B represented the differences of the reconstruction of full data without respiratory gating and multi-bins respiratory gating relative to the reconstruction of first 3 min and 20 s of data, respectively

**Fig. 4:**
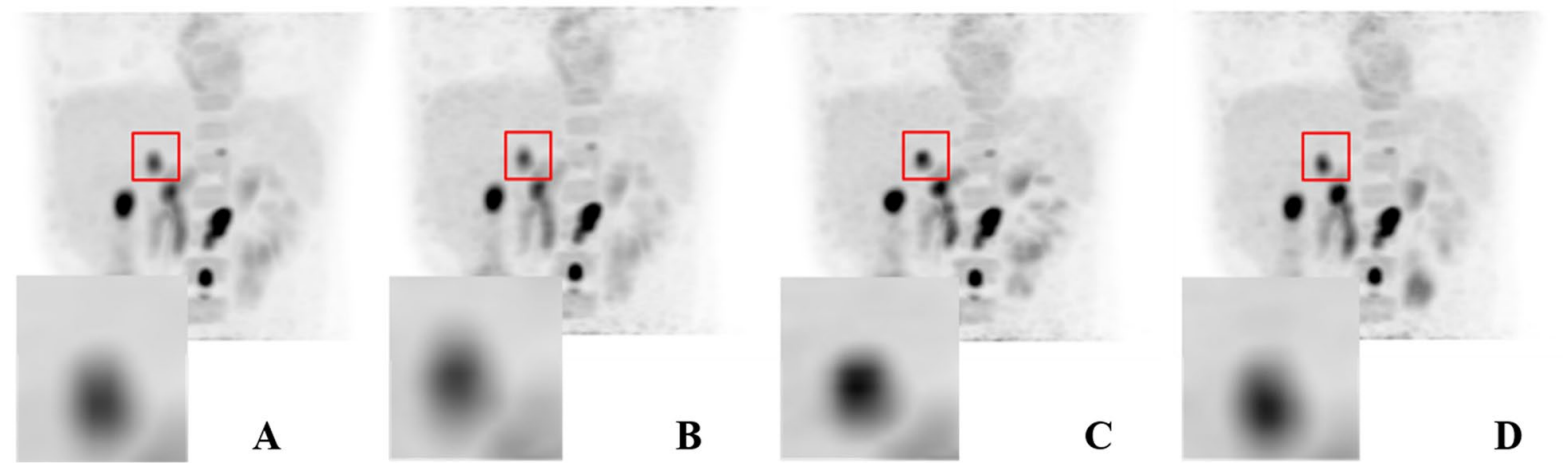
The representative patients’ 3D MIP PET images reconstructed by the four methods, from left to right: reconstruction of full data without respiratory gating (**A**), end-expiratory respiratory gating (**B**), multi-bin respiratory gating (**C**), and reconstruction of the first 3 min and 20 s of data acquired without respiratory gating (**D**). The window centre and width were same for the four images

Figure 4 displays four representative abdomen PET images shown by 3D maximum intensity projection (MIP). The images showed that the lesion was more intense and sharply defined in the image reconstructed by multi-bin respiratory gating than in the other three images, consistent with the statistical results. The background noise in the images reconstructed by reconstruction of full data without respiratory gating was much lower than that in other reconstructed images, which was consistent with the statistical results.

## Discussion

In this study, we evaluated the effects of two respiratory motion correction methods for abdominal PET images obtained by PET/MRI, in which the respiratory correction is considered more important than that in PET/CT. Compared with the reconstruction of the first 3 min and 20 s of data acquired without respiratory gating, the results suggested that multi-bin respiratory gating correction could reduce the PET image blur induced by respiratory motion and increase the SUV_max_ and SUV_mean_ of the lesions significantly, which was much more effective than end-expiratory respiratory gating for abdominal PET/MRI images. However, respiratory motion correction had little effect on the SUV_mean_ of the background, but did increase the background noise and decreased the SNR of PET images because PET counts were discarded.

Although the SUV_max_ of the lesions was significantly higher in the multi-bin respiratory gating group, which was consistent with previous most reports [6, 20–22], the higher SUV_max_ in multi-bin respiratory gating can be traced to two reasons, the results of respiratory motion correction and the increase in image noise with the lower PET counts. The comparisons of different of SUV_max_ in the lesions between the reconstruction of full data without respiratory gating and multi-bin respiratory gating, relative to the reconstruction of the first 3 min and 20 s of data acquired without respiratory gating, did not show significant differences, which suggests that the significant increase of SUV_max_ in lesions after the respiratory motion correction should have relatively high falsely increase induced by the counts reducing. The corresponding analysis was rarely reported by the previous studies. Although the motion artefact of PET images has been reduced after the respiratory motion correction, it probably was that the accuracy of SUVs for the lesions did not improve. Therefore, future studies should develop the respiratory motion correction method without discarding any PET counts.

The multi-bin respiratory gating methods divided the PET data into different phases and used the same phase for reconstruction according to the respiratory cycle. The image blur induced by respiratory motion was reduced and the SUV_max_ and SUV_mean_ for the lesions increased significantly. However, the results of end-expiratory respiratory gating were inconsistent with those of many previous reports [8, 9, 23]. According to the model of end-expiratory respiratory gating as shown in Fig. 1, PET data were collected depending on the amplitude of the respiratory cycle. If the respiratory cycle was not stable and the amplitude decreased irregularly, end-expiratory respiratory gating would reject the counts in the duty cycle. Even it would reject all the PET counts during one respiratory cycle if the amplitude fluctuation deviated largely and maybe resulted in failed tracking during some respiratory cycles, which would affect the PET quality. When the respiratory curve is stable, the lesion in the images from end-expiratory respiratory gating was clearer than that from other methods. In the clinic, we found that the respiratory cycle was often unstable in PET/MRI scanning, which may be because of the following reasons: compared with PET/CT, the bore diameter for the PET/MRI is 10 cm narrower. Second, strong audible noise is always present during the scanning. Third, some MRI sequences during the scanning required patients to hold their breath, which would disrupt the rhythm of respiratory. Therefore, the PET reconstruction from the method of end-expiratory gating has the lowest SNR shown in Table 1, which will decrease the image quality.

There were two parameters for the end-expiratory respiratory gating, offset and width. Results of end-expiratory respiratory gating were also likely related to the parameter settings. To keep the useful time consistent with multi-bin respiratory gating, the offset and width were both set at 33%. The 33% width was also used following a previous recommendation by Grootjans et al., who found that a duty cycle of 35% could provide the best balance between image quality and motion rejection and was thought optimal on ^18^F-FDG PET imaging of lung cancer by PET/CT [12]. However, in a study by Hope et al., the width was also 50%. They found that end-expiratory respiratory-gated liver PET/MRI images increased SUVs and reduced respiratory blurring [23]. Therefore, future studies should further explore the optimal parameters for the end-expiratory respiratory gating for hybrid PET/MRI. The PET attenuation correction during the PET reconstruction was very important for the PET quantitation. In the study, the PET attenuation correction methods for the two respiratory correction methods during the PET reconstruction were same, both based on MRI data and combined tissue segmentation and template matching methods [18]. Therefore, there existed the mismatch between the PET images and the attenuation map induced by motion artefacts, which maybe affects the quantitation of SUVs. In the future studies, the corresponding attention correction methods, which matched the respiratory motion correction PET reconstruction, should also been explored. In this way, the quantitation would be more accurate.

There were some limitations to this study. Firstly, the type of lesions was unknown due to the lack of the underlying physiology, which limit the further investigation about the influence on the clinical diagnosis for the different respiratory correction methods. Meanwhile, the lesions were from different organs, in which the motion artefacts may vary. Secondly, although the results of multi-bin respiratory gating were better than those of end-expiratory respiratory gating with the given parameters, the effect on the fusion of the PET and MRI images was not evaluated in the paper. Meanwhile, the scanning of PET/MRI scanning was usually after PET/CT delayed imaging, so the beginning time of scanning of PET/MRI after the injection of tracer was inconsistent. Finally, the numbers of patients used for the studies was not enough and the statistical deviation probably influenced the results.

## Conclusions

For abdominal PET/MR imaging, the long scanning time makes it more necessary for PET respiratory motion correction than PET/CT. In this study, the results of multi-bin respiratory gating were better than the most common method of end-expiratory respiratory gating with the given parameters. Compared with reconstruction without respiratory correction, multi-bin respiratory gating reduced peripheral blurring of lesions in PET images. It also increased the SUVs of lesions, with two reasons, the respiratory correction and the increasing image noise with the correction. The results suggested there was no significant difference in the increase of SUV_max_ between the two reasons, which was less reported by previous studies. Therefore, multi-bin respiratory gating is proposed as the default reconstruction method for abdominal imaging with PET/MRI. However, the respiratory gating correction with the PET counts loss would also induce the falsely improvement for SUV_max_ of lesions. Meanwhile, the optimal parameters for the end-expiratory respiratory gating in abdominal PET/MRI Imaging should be explored in the future studies.

## Data Availability

The datasets used and/or analysed during the current study are available from the corresponding author on reasonable request.

## Abbreviations

SUV: Standard uptake values
SD: Standard deviation
PET/MRI: Positron emission tomography/magnetic resonance imaging
MRAC: MRI-based attention correction
LAVA: Liver acceleration volume acquisition
